# A systematic review and meta-analysis of canine enteric coronavirus prevalence in dogs of mainland China

**DOI:** 10.1186/s12985-024-02425-8

**Published:** 2024-07-09

**Authors:** Chaoyang Chen, Xiaoxia Ji, Tingting Zhang, Lin Ge, Mengting Sun, Mengting Yang, Cheng Li

**Affiliations:** 1 Department of Medical Science and Technology, Suzhou Chien-Shiung Institute of Technology, Taicang, People’s Republic of China; 2 Jiangsu Province Engineering Research Center of Novel Tumor-Targeting Drug Conjugates, Taicang, People’s Republic of China; 3 Crown Bioscience Inc, Taicang, People’s Republic of China

**Keywords:** CECoV, Systematic review, Meta-analysis

## Abstract

**Background:**

Canine enteric coronavirus (CECoV) is a prevalent infectious disease among dogs worldwide, yet its epidemiology in mainland China remains poorly understood. This systematic review and meta-analysis aimed to assess the prevalence of CECoV in mainland China and identify factors influencing its prevalence.

**Methods:**

A comprehensive literature search was conducted across multiple databases for studies regarding CECoV epidemiology of China. PubMed, CNKI, Wanfang, and CQVIP were searched to obtain the studies. Eligible studies were selected based on predefined criteria, and data were extracted and synthesized. The quality the studies was assessed using the JBI assessment tool. Heterogeneity was checked using I^2^ test statistics followed by subgroup and sensitivity analysis. Subgroup analyses were performed to explore variations in CECoV prevalence by factors such as year, region, season, health status, social housing type, gender, age, and breed. Publication bias was assessed using a funnel plot and eggers test that was followed by trim and fill analysis.

**Results:**

A total of 27 studies involving 21,034 samples were included in the meta-analysis. The overall pooled prevalence of CECoV in mainland China was estimated to be 0.30 (95% CI 0.24–0.37), indicating persistent circulation of the virus. Subgroup analyses revealed higher prevalence rates in younger dogs, multi-dog households, apparently healthy dogs, and certain regions such as southwest China. Seasonal variations were observed, with lower prevalence rates in summer. However, no significant differences in prevalence were found by gender.

**Conclusions:**

This study provides valuable insights into the epidemiology of CECoV in mainland China, highlighting the persistent circulation of the virus and identifying factors associated with higher prevalence rates. Continuous monitoring and surveillance efforts, along with research into accurate detection methods and preventive measures, are essential for the effective control of CECoV and mitigation of its potential impact on animal and human health.

**Supplementary Information:**

The online version contains supplementary material available at 10.1186/s12985-024-02425-8.

## Background

Canine enteric coronavirus (CECoV) is a single-stranded, positive-sense RNA virus within the coronaviridae family, causing mild to severe symptoms in dogs, including diarrhea in adults and systemic symptoms such as vomiting and fever in puppies, especially when concurrent with other gastrointestinal pathogens like parvovirus [1]. Transmission of CECoV occurs through contact with contaminated feces, vomit, saliva, or surfaces [2]. CECoV, an alphacoronavirus, shares genetic recombination history with feline coronavirus (FeCoV) and transmissible gastroenteritis virus of pigs (TGEV) [1]. This recombination accelerates evolution, potentially leading to severe diseases like Feline Infectious Peritonitis Virus (FIPV). Recombination events between CECoV and FeCoV have been hypothesized to give rise to FIPV [3]. Recently, a novel coronavirus, CCoV-HuPn-2018, was isolated from hospitalized children in Malaysia, suggesting cross-species transmission potential from dogs [4]. Additionally, HuCCoV_Z19Haiti was found in a traveler from Haiti, highlighting the risk of CECoV spillover to humans [5]. The possibility of SARS-CoV-2 transmission from humans to pet dogs underscores the role of dogs as potential reservoirs for coronaviruses, with implications for human health [6].

The first CECoV strain, 1–71, was isolated from German military dogs in 1971, marking the beginning of documented CECoV infections worldwide [2]. Studies indicate varying infection rates among diarrheic dogs: 42.1% in Europe [7], 65.5% in Japan [8], and 12.0% in Brazil [9]. In China, CECoV was first identified in 1984, but isolation in mainland China was not official until 1997 [10, 11]. Currently, CECoV infections have been reported in all provinces of mainland China except Hainan and Ningxia provinces. In a prior systematic review, CECoV infection was estimated to have a pooled prevalence of 33% [12]. Furthermore, this review indicated that age, rather than gender, season, or immune status, is associated with CECoV prevalence in Chinese domestic dogs. These data provide a basic reference for our understanding of the epidemiological characteristics of CECoV in China.

However, regional epidemiological studies face limitations due to sample size, sampling location, and seasonal variations in China's diverse climate. Therefore, this systematic review and meta-analysis aim to synthesize CECoV prevalence in mainland Chinese dogs from 1996 to 2022. It also seeks to explore potential risk factors such as geographic region, health status, social housing type, age, gender, season, and breed. This comprehensive analysis aims to enhance understanding of CECoV epidemic patterns and aid in formulating strategies to prevent cross-species transmission.

### Review questions

This systematic review and meta-analysis encompass two main inquiries:

1. Does the pooled prevalence of CECoVs among dogs in Mainland China align with previous literature findings?
2. Does the prevalence of CECoVs in Chinese dogs vary by year, gender, location, season, health status, social housing type, or age?

## Materials and methods

### Search strategy

We conducted a retrospective and documental study following the guidelines of the Preferred Reporting Items for Systematic Reviews and Meta-Analysis (PRISMA) [13]. A meta-analysis protocol was not published prior to this study. Our search strategy involved a comprehensive search on the PubMed database using the subject heading "canine coronavirus" and related terms, including "canine coronaviruses", "coronaviruses, canine", "canine enteric coronavirus", "canine enteric coronaviruses", "dog coronavirus", "dog coronaviruses", "dog enteric coronavirus", "enteric coronavirus, dog", "dog enteric coronaviruses", and "China". Additionally, we searched three Chinese academic databases (CNKI, Wanfang, and CQVIP) for relevant studies using the keywords "canine coronavirus and China" or "canine coronavirus and epidemiology". The search results were imported into Zotero using the PubMed format, and the citation format from the Chinese databases was imported into Zotero using RefWorks. The end date of the search was September 14, 2023.

### Eligibility criteria

The eligibility criteria involved three consecutive evaluations. Firstly, duplicates were removed after importing the search results into Zotero. Secondly, reviewers CC and JX independently assessed titles and abstracts to determine potential usefulness and further select full texts for data extraction. Finally, studies underwent re-evaluation to qualify for meta-analysis and systematic reviews.

Inclusion criteria included: (1) studies on dogs or dog colonies in China; (2) Epidemiological studies focusing on CECoV; (3) studies providing outcome indicators like infection rates or the number of positive cases; (4) studies reporting the specific detection method used; (5) studies providing detailed time and geographical information. Exclusion criteria encompassed: (1) duplicate studies; (2) studies exclusive to foxes, raccoon dogs, or minks; (3) method development studies with a validation sample size less than 10; (4) studies with data integrity issues; (5) literature such as meeting abstracts, case reports, announcements, reviews, or questionnaire-based studies.

### Data extraction

Two independent reviewers, CC and JX, meticulously extracted relevant data from eligible studies. Information such as leading author, publication year, study period, region, design, sample size, positive samples, dog characteristics (age, gender, health status, breeds, social housing), and diagnostic methods were recorded in Excel sheets. Any discrepancies in data extraction were resolved through discussion to ensure consistency. Authors of the studies were not contacted for additional information. Outlier estimates were transformed before analysis to mitigate the effect of high estimates on pooled estimates.

### Quality appraisal

The included literature underwent quality assessment using the Joanna Briggs Institute (JBI) quality appraisal checklist for prevalence studies [14]. Studies deemed to be of low quality were excluded from the meta-analysis.

### Data analysis

Data analysis was conducted using Review Manager 5.4 software. Pooled estimates were generated from the meta-analysis and visualized using Forest plots to illustrate heterogeneity among the included studies. Forest plots summarized estimates with 95% confidence interval (95%CI). Heterogeneity among studies was assessed using Cochrane’s Q test (chi-squared) and Higgins I^2^ statistics. Sensitivity analysis and subgroup analysis were performed to explore potential sources of heterogeneity. Publication bias was evaluated using both subjective (funnel plot symmetry inspection) and objective (Egger's tests) methods. Trim and Fill analysis was utilized to assess the impact of publication bias.

## Results

### Study selection and characteristics

**Fig. 1:**
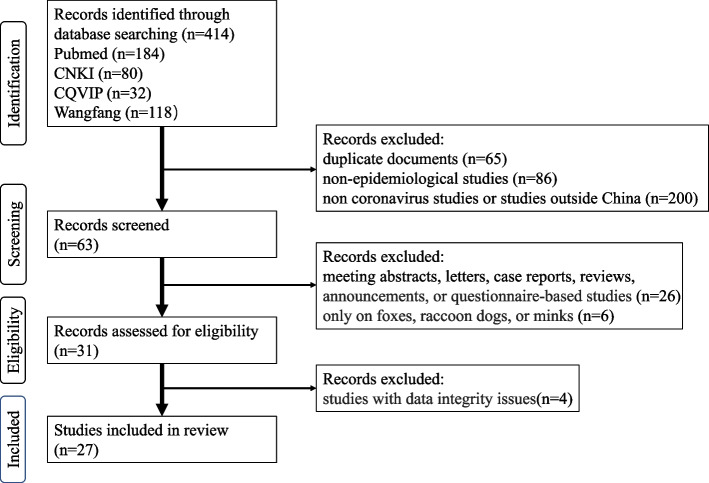
PRISMA study selection flow diagram of included studies

A thorough literature search across Pubmed, CNKI, Wangfang, and CQVIP databases yielded 414 records. Following the removal of duplicates and irrelevant records, 63 papers underwent further screening. Among these, 32 papers were excluded due to their nature as partial results, review papers, or involving animal species not relevant to this study. Ultimately, 27 studies [15–41] were included in the meta-analysis, following the exclusion of 4 papers with data integrity issues (Fig. 1).

In this review, CECoV prevalence was defined as the proportion of CECoV-positive cases to the total number of samples tested. Among the included studies, 25.9% (7/27) were based on data from healthy dogs, while 92.6% (25/27) were based on data from diseased dogs. Similarly, 18.5% (5/27) of the studies were conducted in production colony facilities, whereas 92.6% (25/27) were conducted in veterinary hospitals. Furthermore, 33.3% (9/27) of the studies involved dogs less than 1 year of age, while 29.6% (8/27) involved dogs older than 1 year of age. The majority of the included studies were from eastern and southwestern China. Specifically, 62.96% (17/27) of the studies were cross-sectional in design. Additionally, almost all the included studies were conducted after 2000, except for one study conducted in 1996.

### The pooled prevalence of CECoV in dogs of mainland China

**Fig. 2:**
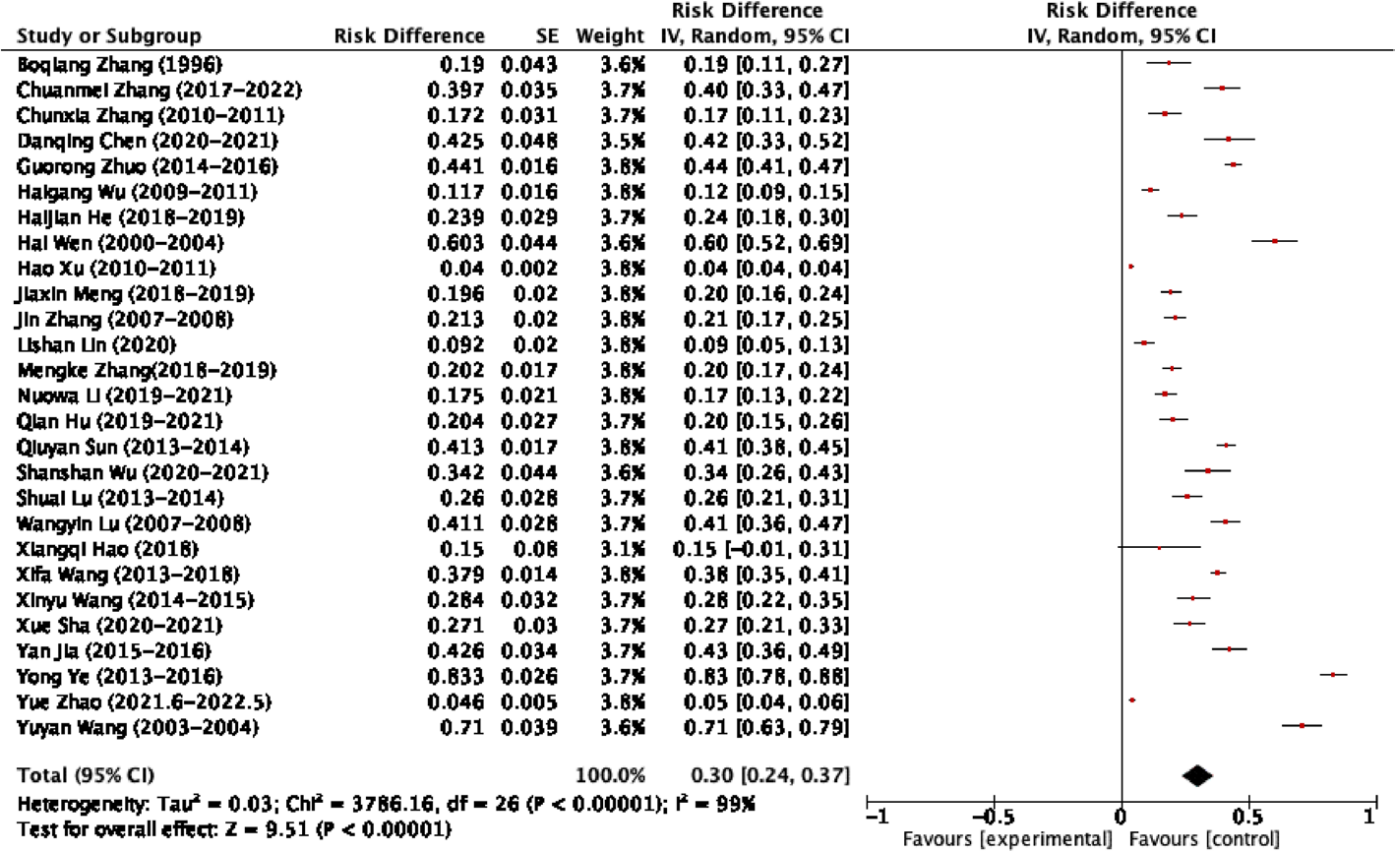
Forest plot of pooled prevalence of CECoV among dogs in mainland China

A total of 21,034 samples, from 27 studies, had been analyzed for the assessment of the pooled prevalence of CECoV (Table 1). According to the subjective assessment of the Galbraith plot (Figure S1), we observed heterogenicity among individual studies. Therefore, we applied a random-effect model for estimating the overall prevalence. Accordingly, the overall pooled prevalence of CECoV was 0.30 (95% CI: 0.24, 0.37). The highest (0.83), and lowest prevalence (0.04), were reported from Central China during 2013.4–2016.3 by Yong Ye [30], and East China during 2010.9–2011.8 by Hao Xu [35], respectively. As shown in the forest plot (Fig. 2), statistically significant heterogeneity was identified (I^2^ = 99%; *p*-value < 0.00001). Due to the significant heterogeneity among the studies, subgroup and sensitivity analyses were done.

**Table 1:** Included studies of CECoV infection among dogs in mainland China

Author	Study period	Province	Region	Sample size	Total positive	Diagnosis method	Study design	Ref
Boqiang Zhang	1996	Jiangsu	East China	84	16	ELISA	Cross sectional	[41]
Hai Wen	2000–2004	Yunnan	Southwest China	35	31	Nested PCR	Cross sectional	[40]
		Jiangsu	East China	80	42			
		Fujian	East China	2	0			
		Guangxi	South China	4	0			
Yuyan Wang	2003.7–2004.2	Jiangsu	East China	73	42	Nested PCR	Surveillance	[39]
		Liaoning	Northeast China	39	34			
		Yunnan	Southwest China	17	15			
		Shanghai	East China	9	7			
Jin Zhang	2007.12–2008.5	Beijing	North China	404	86	Colloidal Gold	Surveillance	[37]
Wangyin Lu	2007.2–2008.12	Gansu	Northwest China	314	129	Colloidal Gold	Cross sectional	[38]
Hao xu	2010.9–2011.8	Shanghai	East China	11,196	448	Colloidal Gold	Surveillance	[35]
Haigang Wu	2009.3–2011.3	Henan	Central China	427	50	Colloidal Gold	Case control	[36]
Chunxia Zhang	2010.6–2011.4	Henan	Central China	151	26	Colloidal Gold	Case control	[34]
Shuai Lu	2013.12–2014.3	Beijing	North China	246	64	RT-PCR	Case control	[33]
Xinyu Wang	2014.5–2015.6	Heilongjiang	Northeast China	201	57	RT-PCR	Surveillance	[31]
Qiuyan Sun	2013.9–2014.9	Shandong	East China	846	349	RT-PCR	Surveillance	[32]
Yong Ye	2013.4–2016.3	Hunan	Central China	198	165	Colloidal Gold	Surveillance	[30]
Yan Jia	2015.3–2016.3	Henan	Central China	209	89	Colloidal Gold	Surveillance	[29]
Xifa Wang	2013.9–2018.8	Guizhou	Southwest China	1233	467	Colloidal Gold	Surveillance	[28]
Guorong Zhuo	2014.3–2016.2	Jiangsu	East China	965	426	Colloidal Gold	Surveillance	[27]
Xiangqi Hao	2018.2–2018.5	Guangdong	South China	20	3	Multiplex PCR	Cross sectional	[26]
Kemeng Zhang	2018.1–2019.1	Jilin	Northeast China	526	106	Colloidal Gold & RT-PCR	Surveillance	[25]
Haijian He	2018–2019	Guangdong	South China	213	51	RT-PCR	Cross sectional	[24]
		Zhejiang	East China					
		Heilongjiang	Northeast China					
		Jiangsu	East China					
		Anhui	East China					
Jiaxin Meng	2018–2019	Heilongjiang	Northeast China	378	74	RT-PCR	Cross sectional	[23]
Danqing Chen	2020.9–2021.3	Jiangsu	East China	106	45	Colloidal Gold & RT-PCR	Cross sectional	[22]
Lishan Lin	2020.3–2020.4	Beijing	North China	7	0	RT-PCR	Cross sectional	[21]
		Chongqing	Southwest China	9	1			
		Jiangsu	East China	113	7			
		Zhejiang	East China	11	0			
		Shanghai	East China	21	4			
		Henan	Central China	20	3			
		Shananxi	Northwest China	14	0			
		Fujian	East China	2	0			
		Anhui	East China	9	4			
Xue Sha	2020–2021	Sichuan	Southwest China	218	59	RT-PCR	Cross sectional	[19]
Chuanmei Zhang	2017–2022	Shandong	East China	199	79	RT-PCR	Cross sectional	[18]
Qian Hu	2019.6–2021.9	Sichuan	Southwest China	216	44	RT-PCR	Cross sectional	[20]
Nuowa Li	2019.11–2021.5	Heilongjiang	Northeast China	325	57	RT-PCR	Surveillance	[17]
		Jilin	Northeast China					
		Liaoning	Northeast China					
		Neimenggu	Northeast China					
Shanshan Wu	2020.11–2021.7	Sichuan	Southwest China	117	40	RT-PCR	Surveillance	[16]
Yue Zhao	2021.6–2022.5	Shandong	East China	1777	81	RT-PCR	Cross sectional	[15]
		Jiangsu	East China

Figure S1 Galbraith plot for subjective assessment of heterogenicity of the pooled prevalence of CECoV

### Subgroup analysis

The studies were stratified based on various factors including study period, geographical area, health status, social housing type, gender, age, season, and breed to identify potential sources of heterogeneity. CECoV prevalence exceeded the overall pooled prevalence of 0.30 in five specific periods, while it was lower in the other six periods (Table 2). The highest prevalence of CECoV was 0.68 (95% CI: 0.62,0.74) during 2003–2004, while the prevalence during 2021–2022 was only 0.05 (95% CI: 0.04, 0.06).

**Table 2:** CECoV prevalence among dogs based on period, region, health status, housing type, gender, age, season, and breed

Subgroups	No. studies	Pooled estimate of CECoV (95%CI)	Heterogeneity: I^2^ (*p*-value)
**Study period**			
1996–1996	1	0.19 [0.11, 0.27]	NA
2003–2004	2	0.68 [0.62, 0.74]	0% (0.41)
2007–2008	2	0.31 [0.12, 0.51]	97% (< 0.00001)
2010–2011	2	0.10 [-0.03, 0.23]	94% (< 0.00001)
2013–2014	3	0.29 [0.15, 0.43]	95% (< 0.00001)
2014–2015	2	0.30 [0.25, 0.35]	0% (0.49)
2015–2016	2	0.41 [0.36, 0.45]	0% (0.49)
2016–2017	1	0.45 [0.39, 0.50]	NA
2017–2018	1	0.40 [0.35, 0.46]	NA
2018–2019	5	0.20 [0.18, 0.23]	0% (0.72)
2020–2021	5	0.26 [0.15, 0.38]	94% (< 0.00001)
2021–2022	1	0.05 [0.04, 0.06]	NA
**Region**			
East China	10	0.31[0.23, 0.39]	99% (< 0.00001)
South China	1	0.15[-0.01, 0.31]	NA
Northwest China	2	0.21[-0.20, 0.61]	98% (< 0.00001)
North China	3	0.20[0.12, 0.28]	77% (0.01)
Central China	5	0.34[0.04, 0.65]	99% (< 0.00001)
Southwest China	7	0.44 [0.29, 0.58]	97% (< 0.00001)
Northeast China	5	0.34 [0.20, 0.47]	97% (< 0.00001)
**Health Status**			
Health	7	0.43[0.18, 0.68]	98% (< 0.00001)
Illness	25	0.30 [0.21, 0.38]	99% (< 0.00001)
**Social housing type**			
Multi-dog	6	0.53 [0.19, 0.87]	99% (< 0.00001)
Single dog	25	0.30 [0.22, 0.38]	99% (< 0.00001)
**Social Housing & Health Status**			
Multi-dog & Health	2	0.84 [0.78, 0.90]	12% (0.29)
Multi-dog & Illness	5	0.42 [0.09, 0.74]	98% (< 0.00001)
Single dog & Health	5	0.26 [0.12, 0.40]	86% (< 0.00001)
Single dog & Illness	25	0.30 [0.22, 0.38]	99% (< 0.00001)
**Gender**			
Male	5	0.31 [0.21, 0.41]	84% (< 0.00001)
Female	5	0.30 [0.21, 0.39]	74% (0.004)
**Age**			
0–3 months	8	0.52 [0.38, 0.67]	93% (< 0.00001)
0–6 months	1	0.50 [0.37, 0.63]	NA
2–7 months	5	0.43 [0.18, 0.67]	98% (< 0.00001)
2–12 months	4	0.39 [0, 0.78]	98% (< 0.00001)
6–12 months	5	0.22 [0.09, 0.36]	82% (0.0002)
> 12 months	8	0.19 [0.11, 0.27]	84% (< 0.00001)
**Season**			
Spring	4	0.27 [0.09, 0.45]	97% (< 0.00001)
Summer	4	0.19[0.01, 0.37]	97% (< 0.00001)
Autumn	4	0.24 [0.07, 0.41]	93% (< 0.00001)
Winter	4	0.24 [0.09, 0.40]	93% (< 0.00001)
**Breed**			
Large breed	3	0.48 [0.12, 0.84]	98% (< 0.00001)
Medium breed	2	0.31 [0.14, 0.48]	90% (0.002)
Small breed	2	0.30 [0.08, 0.53]	96% (< 0.00001)
Mongrel dog	3	0.22 [0.03, 0.40]	96% (< 0.00001)

*CI =* Confidence interval

*I*^*2*^ = Higgins I^2^ statistics

Regional analysis showed varying prevalence, with southwest China exhibiting the highest prevalence (0.44 [95% CI: 0.29–0.58]), followed by central (0.34 [95% CI: 0.04–0.65]) and northeast China (0.34 [95% CI: 0.20–0.47]). Conversely, south China had the lowest rate (0.15 [95% CI: -0.01–0.31]) (Table 2).

Regarding health status, samples from healthy dogs showed a higher prevalence compared to diseased ones, with overall rates of 0.43 (95% CI: 0.18–0.68) and 0.30 (95% CI: 0.21–0.38), respectively. Multi-dog households exhibited a higher prevalence (0.53 [95% CI: 0.19–0.87]) compared to single-dog households (0.30 [95% CI: 0.22–0.38]). Notably, healthy dogs in multi-dog environments had a higher positivity rate (0.84 [95% CI: 0.78–0.90]) compared to diseased ones (0.42 [95% CI: 0.09–0.74]).

Prevalence was higher among dogs under 6 months (0.50 [95% CI: 0.37–0.63]) compared to those older than 12 months (0.19 [95% CI: 0.11–0.27]), with a decrease in prevalence as age increased. Males exhibited a slightly higher prevalence (0.31 [95% CI: 0.21–0.41]) compared to females (0.30 [95% CI: 0.21–0.39]). Seasonal analysis revealed higher prevalence in spring (0.27 [95% CI: 0.09–0.45]) compared to other seasons.

Investigating genetic factors, large breeds showed a higher prevalence (0.48 [95% CI: 0.12–0.84]) compared to medium (0.31 [95% CI: 0.14–0.48]) and small breeds (0.30 [95% CI: 0.08–0.53]). Interestingly, mongrel dogs exhibited a lower positivity rate (0.22 [95% CI: 0.03–0.40]) compared to purebred dogs.

### Sensitivity analysis

Sensitivity analysis demonstrated no significant differences, except for a few outlier studies that deviated from the overall estimate. However, since all studies fell within the 95% confidence interval, the pooled prevalence remained unaffected by individual studies (Figure S2).

Figure S2 Sensitivity analysis on the pooled prevalence of CECoV among dogs in mainland China

### Assessment of publication bias

**Fig. 3:**
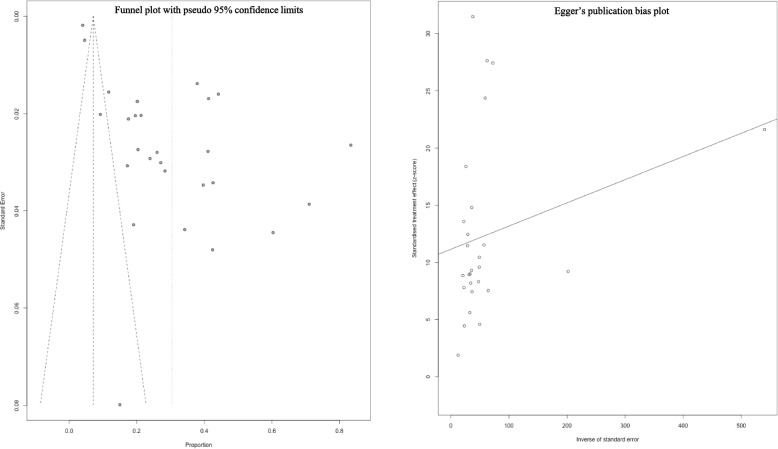
Funnel plot symmetry to check the publication bias (left); Egger's publication bias plot (right)

Evaluation of publication bias through funnel plot analysis revealed evidence of asymmetrical distribution of articles (Fig. 3 left), indicating potential publication bias. Egger's tests further confirmed the presence of publication bias (Fig. 3 right). Subsequently, trim and fill analysis were conducted to illustrate the extent and impact of the publication bias (Figure S3).

Figure S3 A diagram dealing with publication bias applying the trim and fill analysis for the pooled prevalence of CECoV

## Discussion

The study aims to evaluate the overall prevalence of CECoV in mainland China and identify potential factors associated with infection variability. As CECoV remains a commonly encountered infectious disease in dogs, characterized by transient symptoms and limited vaccine availability, understanding its prevalence dynamics is crucial. Recent reports underscore the emergence of mutated and recombinant CECoV strains globally, posing significant threats to both animal and human health [5]. In light of the growing demand for vaccine development and the necessity for preventive and control measures, an updated systematic review is imperative to provide comprehensive insights.

This study conducted a systematic review and meta-analysis of CECoV epidemiological studies conducted between 1996 and 2022, focusing on dogs in mainland China. Analysis of 27 included studies revealed an overall CECoV prevalence of 0.30 (95% CI: 0.24, 0.37), indicating a significant prevalence within mainland China. This underscores concerns regarding the emergence of new CECoV strains and their potential impact on public health security. Our findings closely align with a previous analysis by Dong Bo et al. [12], although some differences exist in inclusion criteria, such as the exclusion of literature due to issues such as unclear study periods and data overlap. Compared to the previous approach employed by Dong Bo et al., this study's search strategy yielded a lower number of hits (414 versus 972). The discrepancy was partially attributed to the omission of Google Scholar, Cochrane Library, and clinical trials databases, which typically produce overlapping results with PubMed. However, the number of screened and included studies was substantially higher in this study (27 versus 15).

Dogs infected with CECoV may experience mild diarrhea, but when accompanied by other pathogens such as CPV, it can result in severe symptoms and possibly death, particularly in young puppies. A key question of interest is whether diseased dogs are more likely to test positive for CECoV. Our subgroup data indicated a higher prevalence of CECoV in healthy than diseased dogs, consistent with the findings of the study conducted by Shanshan Wu et al. during 2020–2021 in Chengdu, China [16]. This may be attributed to healthy dogs being more active and potentially transmitting the virus through social movement. Conversely, environments with more than one dog are associated with a higher positivity rate compared to only one dog.

Given the association between health and multi-dog household with elevated CECoV infection rates, it is imperative to examine the infection rates specifically in healthy dogs residing in multi-dog environments. Our results highlight the potential for increased CECoV prevalence in multi-dog environments and emphasize the importance of regular monitoring for all dogs in such settings. However, it's worth noting that the subset of healthy dogs residing in multi-dog environments is based on only two studies conducted prior to 2003, and the limited data available may impact the reliability of the results. Further research with larger sample sizes and more recent data is warranted to validate these findings.

Dogs of any age can be infected with CECoV, and previous studies have indicated that puppies younger than 6 months of age are at greater risk of infection. Our subgroup analysis investigated the relationship between age and CECoV prevalence, revealing that younger animals have a higher prevalence of infection. While maternal antibodies may offer some protection to puppies, our data suggest that this protection may be limited, and circulating antibodies may not provide sufficient immunity against CECoV infection. However, systematic investigations are needed to assess the effectiveness of circulating antibodies induced by vaccine, although there is currently limited published information on such studies.

For investigating the influence of genetic factors on the likelihood of CECoV infection across different dog breeds. various canine breeds into four categories: large, medium, small, and mongrel dogs. The small breed category includes Chihuahua, Poodle, Pug, Corgi, Shih Tzu, and Bichon Frise. The medium breed category encompasses Border Collie, Huskie, Shiba Inu, Samoyed, and Chow Chow. The large breed category includes German Shepherd, Alaskan Malamute, Golden Retriever, Akita, Great Pyrenee, and Labrador Retriever. Subgroup analysis revealed that the prevalence among medium-sized, small, and mongrel dogs was relatively low, but environmental factors during growth could not be ruled out. Additionally, our analysis found no significant difference in CECoV positivity rates between genders, consistent with previous research findings [16].

This study presents the first analysis of CECoV prevalence distribution over time, revealing two peaks in prevalence in 2003 and 2016–2017, followed by a decline. The decrease in prevalence post-peak could be attributed to various factors, including public health interventions, increased awareness, and advancements in healthcare infrastructure. However, the possibility of episodic events influencing these fluctuations cannot be overlooked. Certain variations in infection rates at different time points may also stem from non-random sample collection practices. For instance, a higher proportion of sick animals or sampling from infected colonies could inflate infection rates. Conversely, research by Zhang Yue on conventional Beagle dog colonies during 2021–2022 reported notably lower infection rates [15]. The excessively low prevalence may also be due to the inapplicability of the assay to emerging mutant viruses.

The diverse climatic conditions across various regions in China likely impact the survival and transmission of CECoV. Previous studies conducted in China have reported varying rates of CECoV infection in different regions. For instance, Tianjin in the north exhibited the highest rate at 60%, followed by Gansu in the northwest at 43%, Shandong in the east at 42%, Beijing in the north at 38%, Heilongjiang in the northeast at 28%, Henan in central China at 24%, Jiangsu in the east at 23%, and Jilin in the northeast at 20% [12]. In our meta-analysis, employing stringent inclusion criteria, the highest prevalence of CECoV was observed in southwest China at 44%, while northern China had a prevalence rate of 20%. However, the limited number of studies available for certain regions, such as south China, may have influenced the results of this study. Despite variations in CECoV prevalence across different regions, no significant difference was observed among these regions.

The climate in China is characterized by continental monsoon patterns, featuring cold winters and hot summers. While no significant regional variations in CECoV prevalence were observed in China, further investigation into potential differences among seasons is essential. Previous surveys conducted in Chengdu revealed that summer had the lowest positive rate (16.0%). However, a systematic review of CECoV infection in Chinese domestic dogs indicated no significant difference between seasons. In our study, no significant difference was found in CECoV prevalence between seasons, and the summer was confirmed to have the lowest prevalence.

Despite these insights, our study encountered significant heterogeneity and publication bias, highlighting the need for cautious interpretation of results. Sensitivity analysis confirmed robustness to outliers, while publication bias may arise from the inclusion of published papers and the diagnostic method. The coronavirus pandemic in the human population has likely sparked heightened interest in researching CECoV epidemiology. However, China currently lacks an official plan for CECoV epidemiology, which may lead to randomized study distribution across different regions and years. While RT-PCR detection was utilized in most studies, it should be noted that certain primer pairs may fail to match the latest CECoV variant, resulting in an underestimation of the positive rate. These circumstances may increase heterogeneity in meta-analyses. Furthermore, the limited number of studies reporting prevalence among healthy dogs underscores the need for further research to understand the full spectrum of CECoV infection.

### Limitations of this review

Firstly, there were variations in sampling time, location, specific dog breeds, sensitivity of the detection methods and housing conditions among the included studies, which may have influenced the results. Pooling the data from these studies regardless of these differences may not fully capture the true variability across different settings. Secondly, the sample sizes in some subgroup analyses were relatively small, which could limit the statistical power to detect significant differences or associations. Thirdly, the use of a single-group analysis in this study may contribute to substantial heterogeneity, as it does not account for potential confounding factors or other sources of variability. Finally, this study did not investigate the impact of immunological and non-immunized factors on the CECoV infection, as most of literatures did not specify the type of vaccine utilized, making it impossible to determine whether the animals were vaccinated against CECoV.

## Conclusion

This systematic review and meta-analysis provide comprehensive insights into the epidemiology of CECoV in mainland China. Through the synthesis of data from 27 studies spanning from 1996 to 2022, our analysis revealed an overall pooled prevalence of CECoV in mainland China of 0.30 (95% CI 0.24–0.37), indicating persistent circulation of CECoV among dogs in the region. Factors such as young age, multi-dog households, and apparently healthy status were associated with higher CECoV prevalence. Regional variations were observed, with southwest China exhibiting a higher prevalence compared to other regions. Additionally, CECoV prevalence was lower in summer and among mongrel dogs, while gender was not found to be associated with prevalence. The continued circulation of CECoV poses a threat to both animal and human health, highlighting the importance of continuous monitoring and epidemiological studies. Moreover, the development of accurate and sensitive detection methods is essential for effective surveillance and control of CECoV in China.

### Supplementary Information

Supplementary Material 1.

Supplementary Material 2.

Supplementary Material 3.

## Data Availability

All relevant data are within the paper and its supporting Information files.

## Abbreviations

CECoV: Canine Enteric Coronavirus
FeCoV: Feline Coronavirus
TGEV: Transmissible Gastroenteritis Virus
FIPV: Feline Infectious Peritonitis Virus
CPV: Canine Parvovirus
RT-PCR: Reverse transcriptase polymerase chain reaction
