# Supplementary figures and images for: A systematic review and meta-analysis of canine enteric coronavirus prevalence in dogs of mainland China
Source: Virol J. 2024 Jul 9;21:155. doi: 10.1186/s12985-024-02425-8 (PMC11234673; doi:10.1186/s12985-024-02425-8)

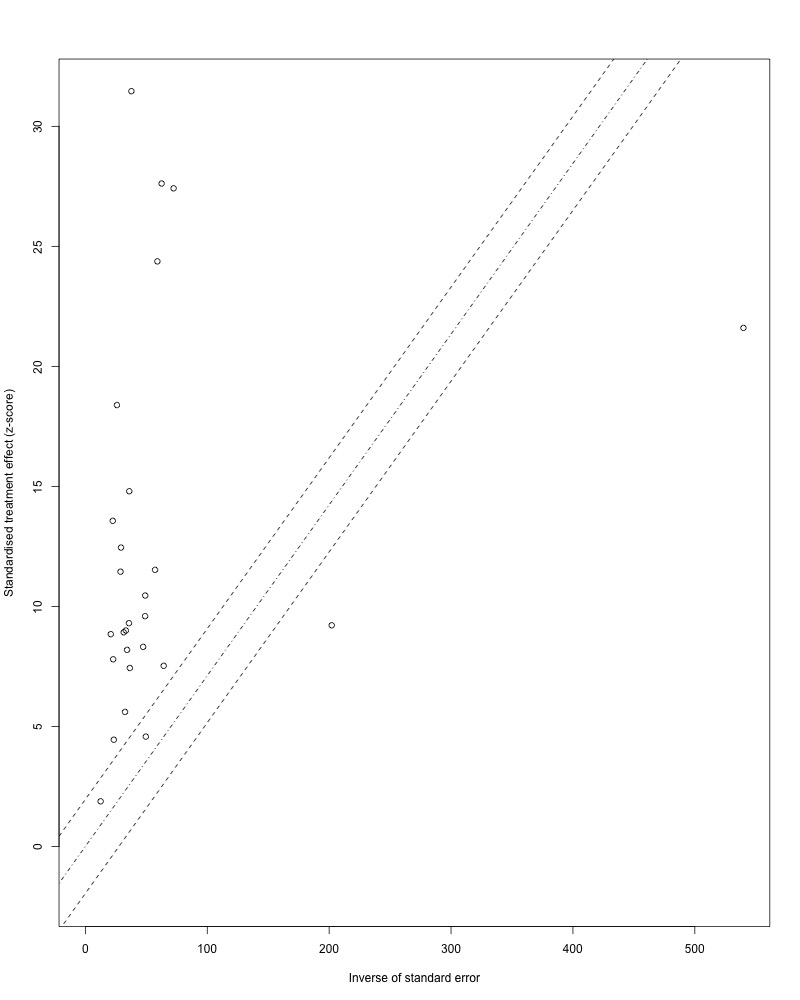

Supplement: Supplementary file 1 — Supplementary Material 1. [file 12985_2024_2425_MOESM1_ESM.jpeg]

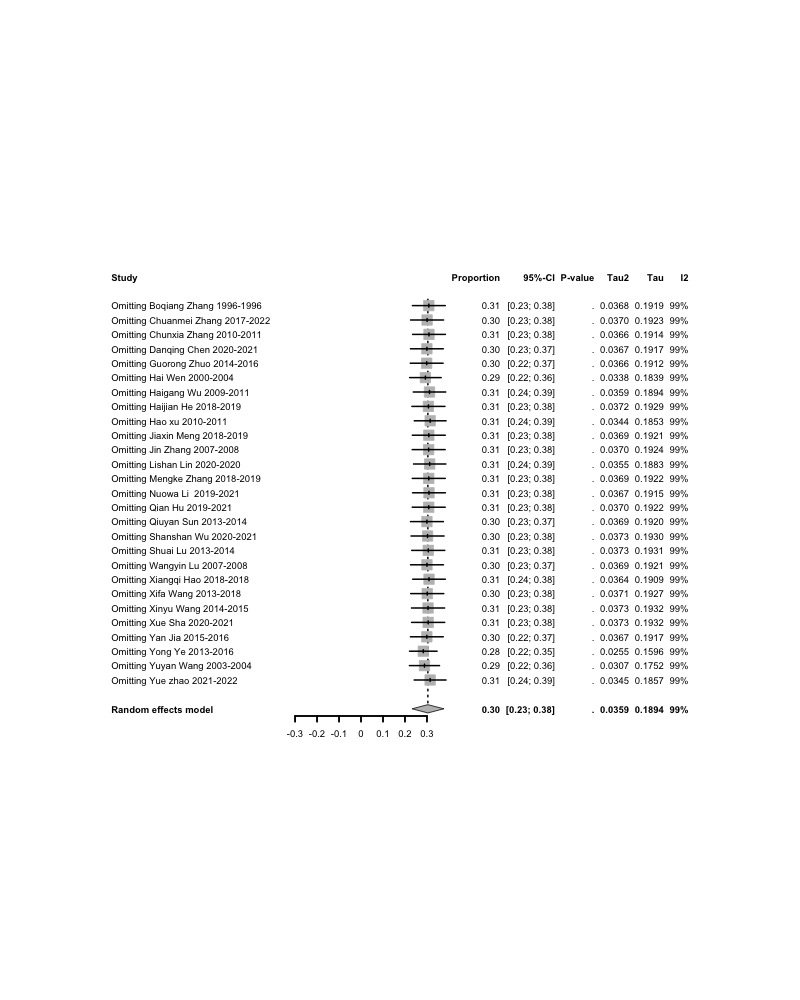

Supplement: Supplementary file 2 — Supplementary Material 2. [file 12985_2024_2425_MOESM2_ESM.jpeg]

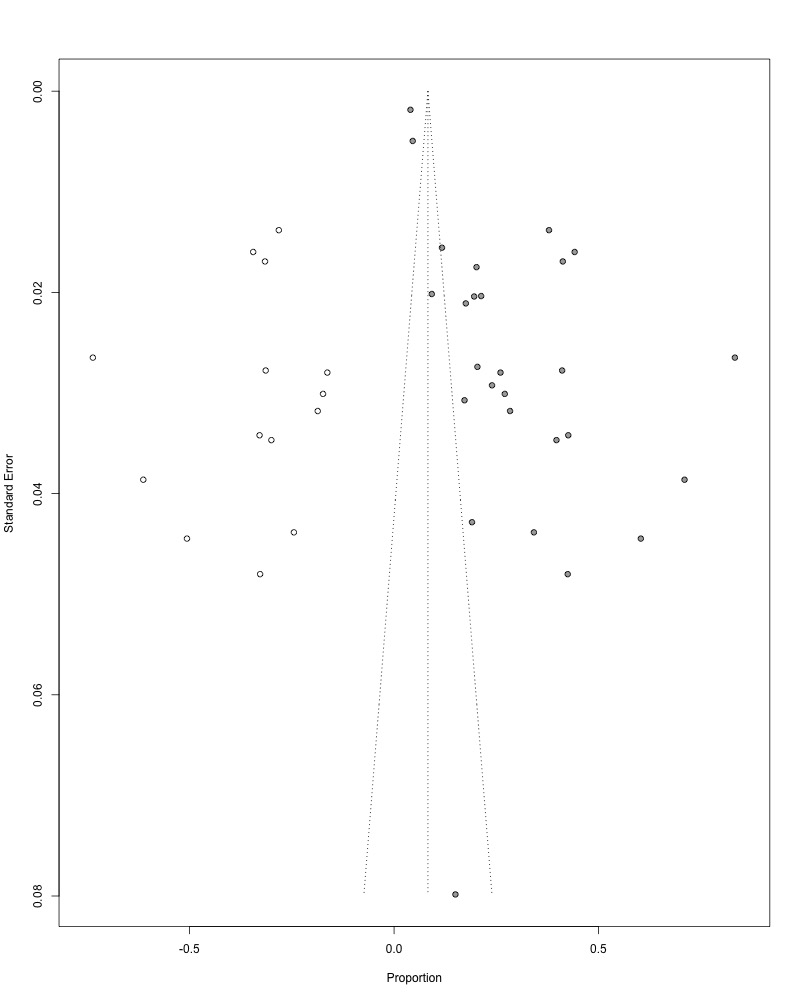

Supplement: Supplementary file 3 — Supplementary Material 3. [file 12985_2024_2425_MOESM3_ESM.jpeg]
